# Paired nerve stimulation with selective compensation effect

**DOI:** 10.3389/fnins.2024.1464336

**Published:** 2024-12-24

**Authors:** Alexey Leukhin, Yuliya Mikhailova, Dinar Masaev, Grigorii Belov, Alexander Toschev, Elsa Fatykhova, Jordi Vallverdú, Max Talanov

**Affiliations:** ^1^B-Rain Labs LLC, Kazan, Russia; ^2^Institute of Information Technologies and Intelligent Systems (ITIS), Kazan Federal University (KFU), Kazan, Russia; ^3^Graz University of Technology, Graz, Austria; ^4^Children's Republican Clinical Hospital of the Ministry of Health of the Republic of Tatarstan, Kazan, Russia; ^5^Catalan Institution for Research and Advanced Studies (ICREA) Academia, Universitat Autònoma de Barcelona, Bellaterra, Spain; ^6^The Institute for Artificial Intelligence R&D, Novi Sad, Serbia; ^7^Department of Engineering, University of Messina, Messina, Italy

**Keywords:** selective inhibition, stimulation, paired associative stimulation (PAS), compensation effect, rehabilitation

## Abstract

**Background:**

In this study we investigate the selective compensation of paired peripheral nerves in healthy humans, focusing on distinct axonal conduction velocities in different fibre types. Using paired associative stimulation (PAS) with adjustable parameters, we aimed to modulate and compensate for neuronal activity along the median nerve.

**Methods:**

Six healthy volunteers (3 male, 3 female, aged: 22–49) participated in the current study. We conducted 30 experiments with the following protocol. A pair of pulses with the following parameters were applied to each volunteer: amplitude, pulse width and inter-pulse delay was generated by the dual-core programmed microcontroller STM32H745xI/G while values were set by one-board computer Jetson Nano. The microcontroller provided a pair of pulses to the DAC that applied it to nerve stimulation sites via a stimulator. During experiments, we used the following ranges: (a) current amplitudes [0–20mA], (b) pulse width [250–500 μs] and (c) delays [50–250 μs]. As the measurement of the stimulation effectiveness, we used the finger's contraction angles.

**Results:**

Our findings reveal a significant selective compensation (inhibitory) effect over the motor responses, demonstrated through variations in finger displacement angles. By optimizing individual parameters-pulse width, inter-pulse delay, and compensatory currents—we successfully induced motor response compensation effects. Notably, consistent compensatory effects were observed across all volunteers using a pulse width of (250 μs) and an inter-pulse delay of (50 μs).

**Discussion:**

These results highlight PAS's potential for developing non-invasive neuromodulation devices. However, further research is required to evaluate its efficacy in individuals with spasticity and upper motor neuron deficits.

## 1 Introduction

Pair Associative Stimulation (PAS) is an innovative approach in neuromodulation that can induce plasticity in the brain through the simultaneous or sequential application of two types of stimulation: peripheral (Ozturk et al., 2023) and central (Chalah et al., 2015). This method has shown significant potential in improving functional connectivity between the cerebellum and the brain, which is important for rehabilitation after stroke and enhancement of motor skills. Considering its ability to modulate neural pathways and enhance motor functions, PAS may have potential applications in addressing neurological disorders that involve motor impairments, such as spasticity. Spasticity is a neurological disorder characterized by a velocity-dependent increase in tonic stretch reflexes with exaggerated tendon reflexes, manifesting as a symptom of upper motor neuron syndrome (Biering-Sørensen et al., 2006). Among the myriad treatment methods, neurosurgical interventions play a pivotal role when conservative methods fail to provide sufficient effect. Currently, there is a vast array of various invasive approaches in treatment, enhancing the efficacy of spasticity therapy (Ayuzawa et al., 2014). However, non-invasive techniques such as PAS and Peripheral Nerve Stimulation (PNS) are gaining attention for their potential to modulate neural pathways without surgical intervention.

The primary mechanisms of Peripheral Nerve Stimulation (PNS) in spasticity treatment include activating large-diameter afferent fibers, which can modulate spinal cord excitability through several pathways (Wilson et al., 2014). One such pathway is the enhancement of presynaptic inhibition using gamma-aminobutyric acid (GABA) to reduce spasticity. Among the various modalities of PNS, Transcutaneous Electrical Nerve Stimulation (tENS) has been extensively studied for its efficacy in treating spasticity (Mills and Dossa, 2016). tENS delivers electrical stimulation through the skin, making it a non-invasive and accessible form of PNS.

The application of PNS in clinical settings, including tENS, has shown promising results in reducing spasticity among various patient groups (Smania et al., 2010). tENS, in particular, is attractive for its ease of use, minimal side effects, and potential to complement or replace more invasive treatment methods.

Studies indicate that repetitive magnetic stimulation could also modulate spinal cord functions, although only a few studies have documented the spasticity-reducing effects induced by this method (Nardone et al., 2015). Moreover, paired peripheral and transcranial stimulation can be used to target the spinal cord and may have the potential for neuromodulation in spinal cord-injured subjects (Kumru et al., 2017). Patients have reported overall improvement and muscle-relaxing effects on affected limbs during stimulation, suggesting a need for further research to determine the effectiveness of this approach.

Although there are not enough studies to completely describe the PAS effect on spinal cord injury (SCI) patients, long-term use of PAS has been found to significantly boost motor functions, with enhancements being more pronounced on the PAS-treated side (Versace et al., 2018). PAS is targeted to stimulate and maintain neuroplasticity to recover axonal connections and strengthen synaptic connections. These observations correlate with clinical improvements, especially significant a month after the start of the intervention, confirming the long-term positive effect of PAS on recovery after SCI (Vanhanen et al., 2022).

Ongoing research is focused on determining the optimal stimulation parameters, including pulse width and inter-pulse delay, to enhance the therapeutic benefits of PAS in spasticity treatment. There is growing interest in the combined effects of PAS and other treatment methods, such as pharmacological interventions and botulinum toxin injections, for a comprehensive approach to spasticity management (Hok et al., 2021).

This is particularly relevant given the significant challenges posed by spasticity, a prevalent symptom of upper motor neuron syndrome. Its debilitating impact on motor function and quality of life underscores the need for advanced, integrated therapeutic approaches (Trompetto et al., 2014). Despite the availability of various treatment modalities, achieving effective long-term management of spasticity remains a critical unmet need in clinical practice (Morone et al., 2023). Advances in PAS and its combination with other modalities offer a promising path forward, addressing these challenges through innovative and individualized therapeutic strategies.

In response to this challenge, innovative neuromodulation techniques such as PAS have emerged as promising avenues for inducing neuroplasticity and improving motor outcomes in individuals with neurological disorders. By concurrently stimulating peripheral and central nervous system components, PAS offers a unique approach to enhancing functional connectivity and facilitating motor recovery (Hartwigsen and Volz, 2021). In addition to spasticity management, our study extends the application of PAS technology to the development of a selective neuronal activity compensation device. This device utilizes PAS with adjustable parameters, including amplitude, pulse width, and inter-pulse delays, to trigger compensatory neural activity in targeted motor pathways.

In the subsequent sections of this paper, we will explore the methodology employed in our study, present our empirical findings in detail, discuss the implications of our results for clinical practice and future research directions, and conclude with a reflection on the broader significance of PAS in the field of neurological rehabilitation.

## 2 Subjects and methods

### 2.1 The system architecture

**Figure 1:**
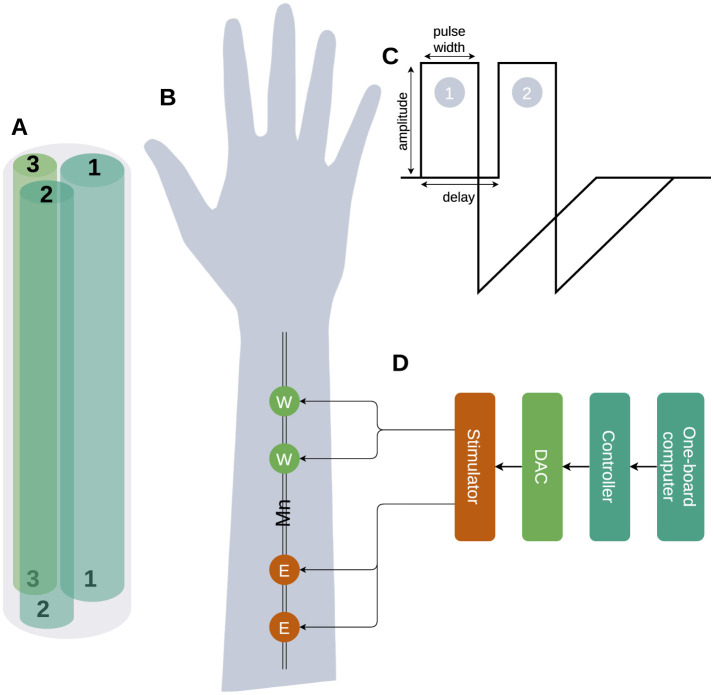
The high-level diagram of the selective compensation device. **(A)** The schematic picture of a median nerve with only three fibers is shown with different parameters: width, R, C, and number of Na/K channels. **(B)** The stimulation site Elbow (E) triggers a neuronal activity distribution along with the fiber and the reading and compensation Wrist (W) site is used to read the neuronal activity triggered by the stimulation to detect the delay between stimulation pulses and distributed neuronal activity and later compensation of triggered activity. **(C)** Triggering (1) applied to site E and compensation (2) applied to site W pulses. **(D)** The stimulation device architecture.

In this work, we propose an approach focused on the precise PAS to trigger and later compensate for the distributed neuronal activity along the nerve. The median nerve consists of several axons and dendrites with different (individual) parameters: size, resistance (R), capacitance (C), myelination, and conductance of sodium and potassium channels (Figure 1A; Ahmed et al., 2022). The transcutaneous electrical stimulation current triggers the neuronal activity in fibers and it is influenced by: the value of a stimulation current, fiber depth, myelination and threshold value etc (Talanov et al., 2021). After the stimulation with pulse 1 depicted in Figure 1C near an elbow (Figure 1B site E), the triggered neuronal activity distributes along the fibers of a medial nerve with different speeds identified by capacitance, resistance, sodium and potassium channels density, threshold voltage and myelination of each particular fiber (Figure 1A), thus triggered spikes in site E (Figure 1B) reach the wrist site (W) at different specific moments. Knowing the precise moments of the spikes' arrival we can compensate for the subthreshold fiber membrane potentials with a compensatory pulse (Figure 1C2) to prevent the fiber from further distributing the neuronal activity. Simplified example: fibers 1, 2, 3 have individual resistance R1, R2, and R3 (Figure 1A) where *R*1 < *R*2 < *R*3, thus neuronal activity distribution delays are *d*1 < *d*2 < *d*3; generating compensatory pulses at moments d1 and d3 we could filter the distribution of the neuronal activity along fibers 1 and 3 letting spikes pass through fiber 2.

A pair of pulses with the following parameters: amplitude, pulse width and inter-pulse delay (Figure 1C) was generated by the dual-core programmed microcontroller STM32H745xI/G while values were set by one-board computer Jetson Nano. The microcontroller provided a pair of pulses to the DAC that applied it to nerve stimulation sites (E, W) via stimulator (Figure 1D). During experiments, we used the following ranges: (a) current amplitudes [0–20 mA], (b) pulse width [250–500 μs] and (c) delays [50–250 μs]. As the measurement of the stimulation effectiveness, we used the finger's contraction angles.

### 2.2 Assumptions

In the current work, we used the following assumptions:

1. **Assumption 1**: Our approach involves using two stimulation sites (Figure 1B) on the median nerve: the elbow (E) and the wrist (W), both connected to a single stimulator managed by a microcontroller and a one-board computer. When a stimulation pulse is applied to site E, it triggers neuronal activity that propagates along the nerve fibers toward site W. By carefully setting the pulse width and delay parameters, we can apply a compensatory pulse at site W precisely timed to compensate the neuronal activity initiated at site E. This setup aims to inhibit or compensate for the neuronal activity at site W, effectively filtering out specific nerve signals based on their propagation characteristics.
2. **Assumption 2**: the setup step of delay (50 μs) and pulse width (250 μs) is small enough to match the individual parameters of neuronal activity propagation along the medial nerve of each volunteer (Figure 1C).
3. **Assumption 3**: the temporal parameters of the stimulation, including the pulse width and inter-pulse delay, are precise enough to selectively activate a specific fiber within the median nerve of a particular research participant.

The hypothesis we checked: *we should observe the compensatory effect of the triggering pulses (E) via compensatory pulses (W) with individual per volunteer setup of pulse width and inter-pulse delay parameters*.

### 2.3 Validation

1. We have validated the two sites paired synchroniaed stimulation and demonstrated a compensatory effect with all volunteers with individual pairs of parameters: pulse width and inter-pulse delay (see Section 3).
2. During our experiments we demonstrated that we could trigger a compensatory effect in every volunteer with the pair of steps inter-pulse delay (50 μs) and pulse width (250 μs) (see Section 3).
3. We failed to trigger specific sensations in a particular research participant's medial nerve and demonstrated only the nerve stimulation's overall inhibitory/compensatory effect.

### 2.4 Research involving humans and animals statement

Six healthy volunteers participated (three male, three female, age: 22–49) in the current study.

### 2.5 Informed consent

All participants gave informed written consent to participate in the study, in accordance with the Declaration of Helsinki, and were introduced to the study protocol.

## 3 Results

We studied the inhibitory effect produced by the paired synchronized stimulation median nerve in elbow and wrist sites. Firstly we recorded the angle of the finger displacement with no stimulation, then we stimulated the elbow site (E) (Figure 1B) until we could register visible motor response initiation and recorded the angle. Later we stimulated the wrist site (W) with variable delays in the range described above. We observed two variations of the paired stimulation effect: (1) *compensation* finger displacement angle decreased and (2) *summation* finger displacement angle increased.

We conducted 30 experiments per volunteer and recorded changes in voluntary muscle contraction angles during the stimulation experiment for this we recorded the following angles: (1) no stimulation angle, (2) elbow stimulation angle, and (3) wrist stimulation angle and calculated Δ*angle* = (*ESA*−*NSA*)−(*WSA*−*NSA*); where *ESA* stands for the angle of muscle contraction with only elbow stimulation, *WSA* – wrist stimulation angle of muscle contraction, *NSA* – no stimulation angle (baseline). Later we selected experiments with significant compensatory effects.

**Figure 2:**
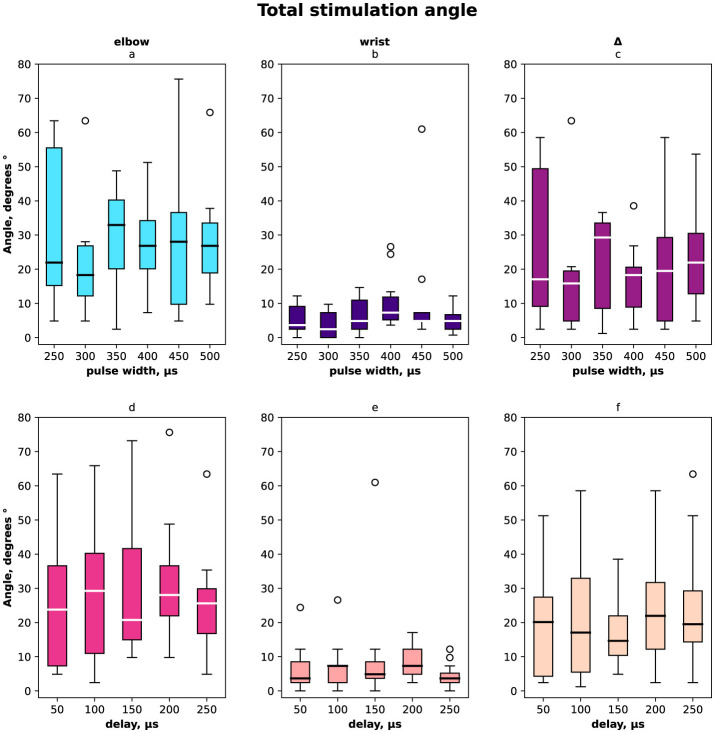
Box-plot of selective compensation effect measured as finger displacement angles for both females and males. The solid line is the mean and circles represent outliers. **(A)** Angles for the range of pulse width in μs of the elbow stimulation. **(B)** Displacement angles for the range of pulse width in μs of the elbow and wrist stimulation. **(C)** Δ between angles before and after compensatory current application to wrist site for the range of pulse width in μs. **(D)** Angles for the range of delays in μs of the elbow stimulation. **(E)** Displacement angles for the range of delays in μs of the elbow and wrist stimulation. **(F)** Δ between angles before and after compensatory current application to wrist site for the range of delays in μs.

**Figure 3:**
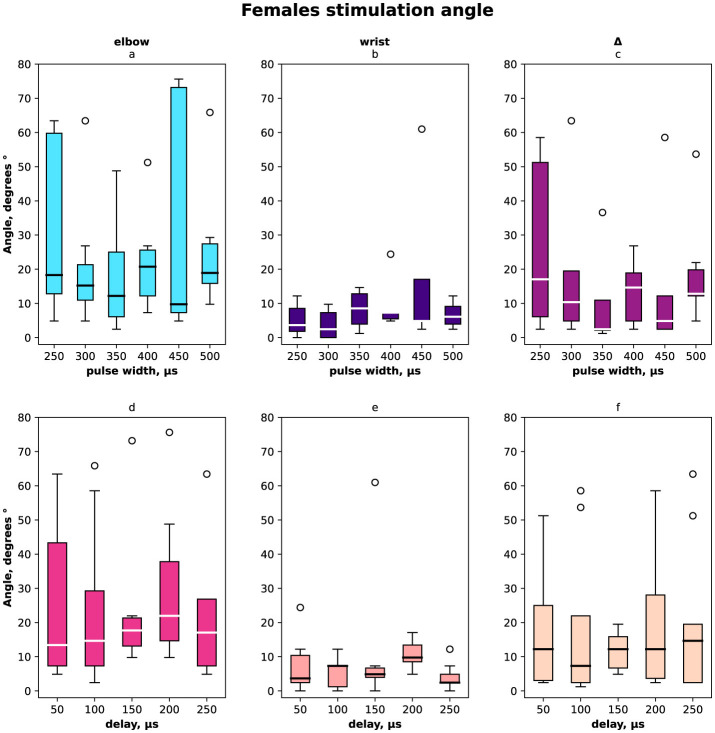
Box-plot of selective compensation effect measured as finger displacement angles for females only. The solid line is the mean and circles represent outliers. **(A)** Angles for the range of pulse width in μs of the elbow stimulation. **(B)** Displacement angles for the range of pulse width in μs of the elbow and wrist stimulation. **(C)** Δ between angles before and after compensatory current application to wrist site for the range of pulse width in μs. **(D)** Angles for the range of delays of the elbow stimulation. **(E)** Displacement angles for the range of delays of the elbow and wrist stimulation. **(F)** Δ between angles before and after compensatory current application to wrist site for the range of delays.

**Figure 4:**
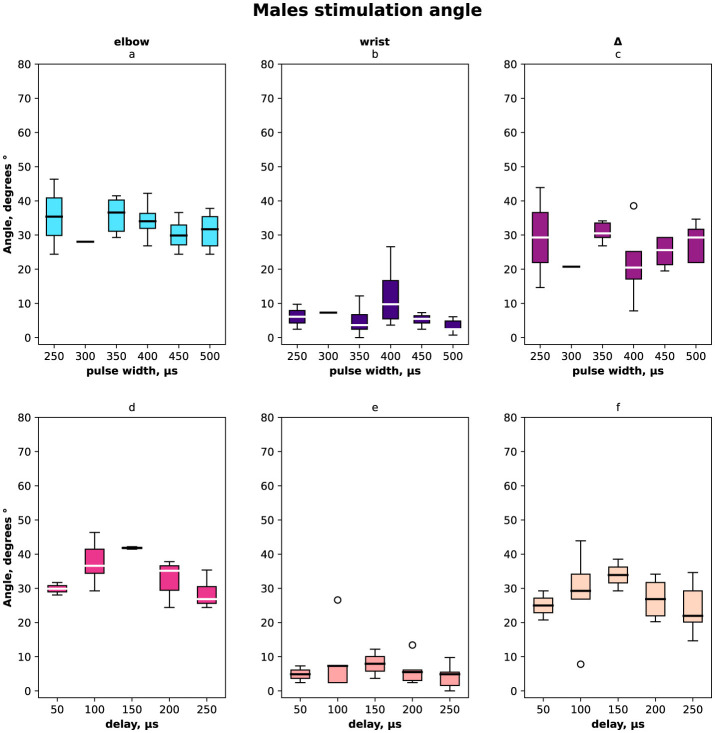
Box-plot of selective compensation effect measured as finger displacement angles for males only. **(A)** Angles for the range of pulse width in μs of the elbow stimulation. **(B)** Displacement angles for the range of pulse width in μs of the elbow and wrist stimulation. **(C)** Δ between angles before and after compensatory current application to wrist site for the range of pulse width in μs. **(D)** Angles for the range of delays of the elbow stimulation. **(E)** Displacement angles for the range of delays of the elbow and wrist stimulation. **(F)** Δ between angles before and after compensatory current application to wrist site for the range of delays.

The distribution of angles *ESA, WSA* with regards to pulse width and delays are shown in Figure 2 for females and males together, in Figure 3 for females only and in Figure 4 for males where (a, d) is the elbow stimulation angle when we can register the motor response initiation, (b, e) the finger displacement angle for both wrist and elbow stimulation with compensatory effect registered, (c, f) the difference between stimulation only elbow and stimulation at both sites; (a–c) according to a pulse width, (d–f) according to a delay.

**Figure 5:**
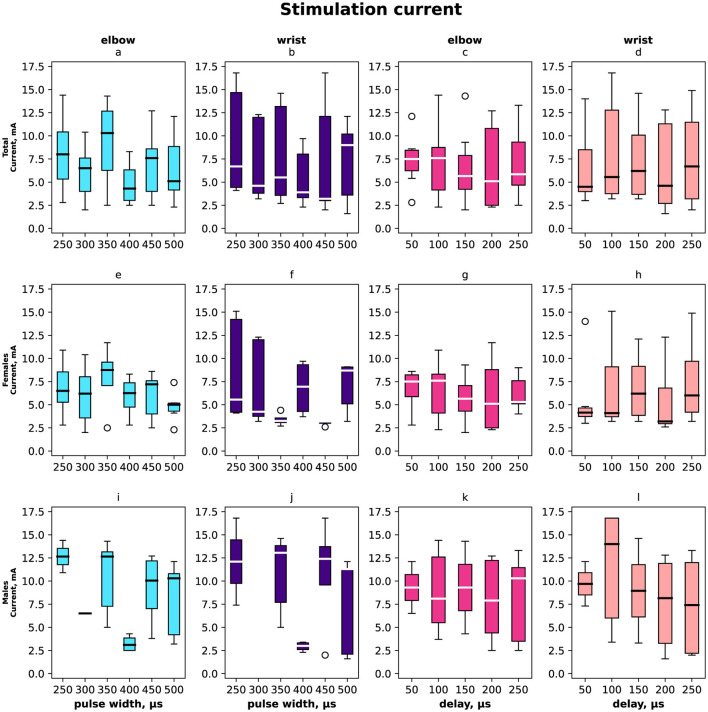
Box-plot of stimulation currents of the elbow and wrist for the selective compensation effect. The solid line is the mean and circles represent outliers. **(A, E, I)** The minimal elbow stimulation current for motor response in mA with the range of pulse widths in μs overall, females, and males. **(B, F, J)** The minimal current that elicits a compensation effect for wrist stimulation in mA with the range of pulse width in μs. **(C, G, K)** Selective compensation effect currents in mA applied to elbow with the range of delays in μs. **(D, H, L)** Selective compensation effect currents applied to wrist with the range of delays between stimulations in μs.

The stimulation current varied from 2.5 mA to 17.5 mA and its distribution shown in Figure 5 (a–d) total distribution, (e–h) distribution for females and (i–l) for males, (a, e, i, c, g, k) for elbow site, (b, f, j, d, h, l) for wrist site, (a, e, i, b, f, j) according to pulse width, (c, g, k, d, h, j) according to delay. The minimal current was registered for the paired stimulation compensatory effect with pulse width 400 μs in total and the same value for males as for females it was 350 μs and 450 μs.

In total and for females the elbow stimulation angle varied from 5° to 75° (Figures 2, 3). For males the elbow stimulation angle varied from 25° to 45° (Figure 4). The angle with compensation stimulation varied from 0° to 15° (Figure 2). For females the compensated angle varied from 0° to 15° (Figure 3). For males the compensated angle varied from 0° to 30° (Figure 4). We registered the highest finger displacement angle with pulse width 250 μs, delays 50 μs and 200 μs. The finger displacement angle for elbow stimulation of females had a higher variance than for males (Figures 3, 4). In total and for females the most effect of compensation was on 250 μs and 350 μs pulse width with no significant dependency on delay (Figures 2, 3). For males the most effect of compensation was on 250 μs and 350 μs pulse width and on 150 μs delay (Figure 4).

On average, the compensatory effect for males was higher than for females. It is represented in Δ between angles before and after compensatory current application, for males median Δ 25 ± 5° while for females 15 ± 10°.

For further details about Δ between angles before and after the application of compensatory current to the wrist site for different pulse width and delay conditions refer to Table 1. N/A indicates no compensatory effect for specific volunteer on specific pulse width and delay.

**Table 1:** Δ between angles before and after the application of compensatory current to the wrist site for different pulse width and delay conditions.

**Pulse width, μs**	**Delay, μs**	**Volunteer 1**	**Volunteer 2**	**Volunteer 3**	**Volunteer 4**	**Volunteer 5**	**Volunteer 6**
250	50	—	—	—	51.2°	—	19.5°
250	100	43.9°	—	—	58.5°	7.3°	2.4°
250	150	—	—	—	—	—	—
250	200	14.6°	—	—	—	—	—
250	250	—	—	—	51.2°	2.4°	14.6°
300	50	20.7°	—	—	—	4.9°	—
300	100	—	—	—	—	4.9°	—
300	150	—	—	—	—	4.9°	19.5°
300	200	—	—	—	—	2.4°	19.5°
300	250	—	—	—	63.4°	—	15.9°
350	50	—	—	—	—	—	—
350	100	26.8°	34.1°	—	—	1.2°	—
350	150	—	29.3°	—	36.6°	—	—
350	200	34.1°	31.7°	—	—	2.4°	—
350	250	—	29.3°	—	—	2.4°	—
400	50	—	—	—	26.8°	2.4°	—
400	100	7.8°	—	—	—	2.4°	12.2°
400	150	38.5°	—	—	—	—	17.1°
400	200	20.2°	—	—	—	—	—
400	250	20.7°	—	—	—	—	19.5°
450	50	—	—	—	12.2°	2.4°	—
450	100	—	—	29.3°	—	—	—
450	150	—	—	—	—	—	—
450	200	—	21.9°	—	58.5°	4.9°	—
450	250	29.3°	19.5°	—	—	2.4°	—
500	50	—	29.3°	—	—	4.9°	—
500	100	—	—	—	53.7°	—	22.0°
500	150	—	—	—	—	—	12.2°
500	200	31.7°	21.9°	—	—	—	12.2°
500	250	34.6°	21.9°	—	—	—	13.4°

Volunteers 1–3 are male participants, and Volunteers 4–6 are female participants. Dash sign (“—”) indicates no compensatory effect observed for the corresponding pulse width and delay condition.

**Figure 6:**
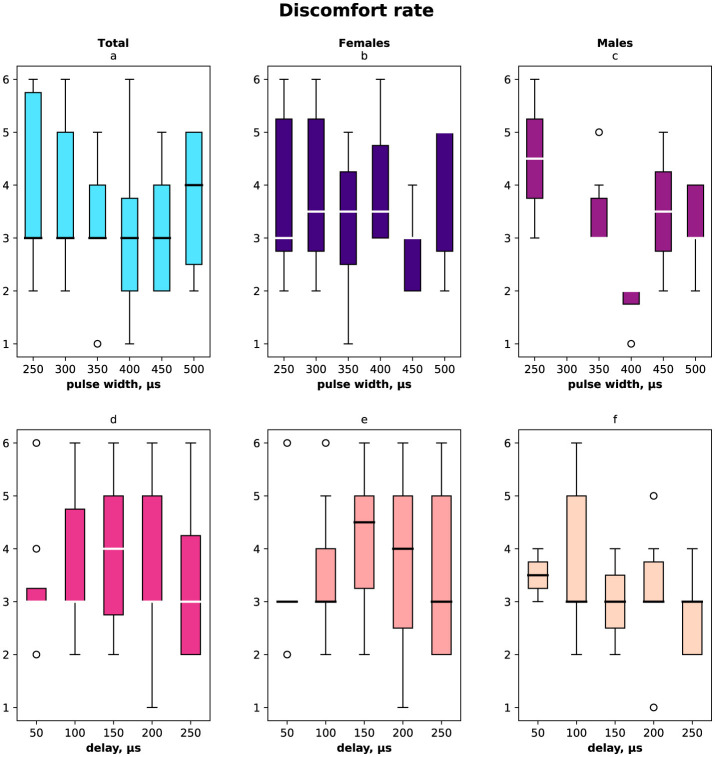
Subjective discomfort rate due to the stimulation measured in the range [1…10]. **(A)** Discomfort rate measured for both females and males for the range of pulse width in μs. **(B)** Female discomfort rate for the pulse width range. **(C)** Male discomfort rate for the pulse width range. **(D)** Discomfort rate for both females and males for the range of delays in μs. **(E)** Female discomfort rate in the range of delays. **(F)** Male discomfort rate in the range of delays.

The registered subjective discomfort rate is shown in Figure 6 (a, d) total distribution, (b, e) female distribution, and (c, f) male distribution regarding pulse width (a–c) and delay (d–f). The discomfort rate was measured subjectively in the range from 1 to 10 where 10 was the highest discomfort. We noted that male discomfort rate distribution had less variance than females. The minimum discomfort rate for males was with stimulation pulse width 400 μs, delay 250 μs, for females minimum was registered with stimulation pulse width 450 μs, delay 50 μs.

## 4 Discussion

The present study demonstrates the potential for selective compensation of motor nerve fibers through conduction velocity-dependent filtering by applying paired median nerve stimulation in healthy volunteers. The subject cohort comprised six participants (three male, three female) aged 22–49 years. A total of 30 experiments per volunteer were conducted, and the effect was quantified by the change in voluntary contraction angle. As a result, we successfully achieved a compensatory effect in 100% of the volunteers, adjusting for individual delays and pulse widths.

The results provide preliminary evidence that mixed peripheral nerves may be effectively neuromodulated in a fiber-specific manner based on axonal conduction velocities. The proposed multi-focal paired stimulation paradigm offers a novel non-invasive approach for targeting particular motor axons. These findings highlight the prospects of peripheral nerves as conduits for precisely timed, distributed bio-electronic therapies. However, the small sample size limits the generalizability of these findings, and future studies with larger, more diverse populations are necessary to validate these results across clinical contexts (Versace et al., 2018).

The integration of medicine, engineering, and cognitive philosophy through the development and application of PAS holds significant social and clinical implications. The research presented in this paper not only advances scientific understanding but also offers tangible benefits that can reshape healthcare delivery, improve patient outcomes, and contribute to societal wellbeing. One of the most immediate social impacts of this research is the potential improvement in the quality of life for individuals suffering from neurological disorders, particularly those with spasticity and spinal cord injuries. By offering a non-invasive, effective method for managing spasticity and promoting motor recovery, PAS can alleviate the physical and psychological burdens associated with these conditions. Patients can experience greater independence and a reduction in pain and discomfort, leading to enhanced overall wellbeing and a more active, fulfilling life (Trompetto et al., 2014).

Despite the promise of PAS, its potential applications should be compared against standard treatments for spasticity, such as botulinum toxin injections and neurosurgical interventions, to better understand its relative efficacy, cost-effectiveness, and safety. While PAS is highlighted as non-invasive and cost-efficient, a detailed comparative analysis would help solidify its clinical positioning (Morone et al., 2023). Moreover, addressing the discomfort experienced by participants during stimulation, as observed in the current study, will be crucial for enhancing patient compliance and broadening clinical adoption (Vanhanen et al., 2022).

The implementation of PAS as a therapeutic intervention also has the potential to reduce long-term healthcare costs. Traditional treatments for spasticity often involve expensive and invasive procedures, prolonged hospital stays, and continuous use of medication (Biering-Sørensen et al., 2006). PAS, with its non-invasive nature and effectiveness, can decrease the need for such extensive medical interventions. This reduction in healthcare resource utilization can lead to significant cost savings for both healthcare providers and patients, making high-quality care more accessible and sustainable.

The development of wearable stimulaters and programmable pulse generators as part of PAS technology represents a significant step toward personalized medicine. These advancements allow for treatment plans tailored to the specific needs of each patient, optimizing therapeutic outcomes. Personalized medicine not only enhances the efficacy of treatments but also empowers patients by involving them more directly in their care. Patients can manage and adjust their therapy parameters, leading to a greater sense of control and engagement in their health management (Guidali et al., 2021).

Further, improvements in the treatment of neurological disorders can have positive economic implications. As patients experience better recovery outcomes, they are more likely to return to work and contribute to the economy. This can alleviate some of the financial strain associated with disability and long-term care. Additionally, the development and deployment of PAS technology can create new job opportunities in the fields of biomedical engineering, healthcare, and rehabilitation services (Smania et al., 2010).

The integration of cognitive philosophy in PAS research brings to light important ethical and philosophical considerations. The ability to generate artificial sensations, such as temperature and weight perception, raises questions about the nature of human experience and the potential for enhancing or altering sensory perception. Ethical considerations, including patient autonomy, informed consent, and safeguards against misuse, must be addressed to ensure PAS technology is developed and applied in ways that respect human dignity and autonomy (Hartwigsen and Volz, 2021).

Finally, the interdisciplinary nature of this research fosters collaboration between medicine, engineering, and cognitive sciences, creating rich educational and research opportunities. Universities and research institutions can develop new curricula and research programs focused on neuromodulation and its applications. This can lead to the training of a new generation of scientists and engineers equipped to tackle complex healthcare challenges, further driving innovation in the field. Therefore, we can affirm that the social and clinical impact of PAS research extends far beyond the scientific realm. By improving patient outcomes, reducing healthcare costs, empowering individuals, and fostering interdisciplinary collaboration, PAS has the potential to bring about significant positive changes in society. As we continue to explore and develop this promising technology, it is essential to consider and address the broader social and ethical implications to maximize its benefits for all.

## 5 Limitations

The presented method has the following limitations: (1) the step of the inter-pulse delay setup should be < 50 μs the finer the step the wider ranges of compensatory effects we can observe; (2) the pulse with steps is 50 μs the less sensitive setup of the experiment though it still has a significant impact on the ranges of compensatory effects; (3) individual parameters of hands including linear size and muscle volume and depth of nerves have the significant impact on the delay/pulse width combination.

## Data Availability

The raw data supporting the conclusions of this article will be made available by the authors, without undue reservation.
